# Hydrolysate from Eggshell Membrane Ameliorates Intestinal Inflammation in Mice

**DOI:** 10.3390/ijms151222728

**Published:** 2014-12-09

**Authors:** Yaning Shi, Prithy Rupa, Bo Jiang, Yoshinori Mine

**Affiliations:** 1 State Key Laboratory of Food Science and Technology, School of Food Science and Technology, Jiangnan University, Wuxi 214122, China; E-Mails: yanings@uoguelph.ca (Y.S.); bjiang@jiangnan.edu.cn (B.J.); 2 Department of Food Science, University of Guelph, 50 Stone Road East, Guelph, ON N1G 2W1, Canada; E-Mail: pbabu@uoguelph.ca

**Keywords:** eggshell membrane hydro lysate, inflammatory bowel disease, DSS (dextran sodium sulphate)-induced colitis, pro-inflammatory cytokines

## Abstract

Inflammatory bowel diseases (IBD) comprises of ulcerative colitis (UC) and Cohn’s disease (CD) as two main idiopathic pathologies resulting in immunologically mediated chronic inflammatory conditions. Several bioactive peptides and hydro lysates from natural sources have now been tested in animal models of human diseases for potential anti-inflammatory effects. Eggshell membrane (ESM) is a well-known natural bioactive material. In this study, we aim to study the anti-inflammatory activity of ESM hydro lysate (AL-PS) *in vitro* and *in vivo*. *In vitro*, AL-PS was shown to inhibit pro-inflammatory cytokine IL-8 secretion. *In vivo* treatment with AL-PS was shown to reduce dextran sodium sulphate (DSS)-induced weight loss, clinical signs of colitis and secretion of interleukin (IL)-6 (*p* < 0.05). In addition, treatment with AL-PS also attenuated the severity of intestinal inflammation via down-regulation of IL-10 an anti-inflammatory cytokine. This validates potential benefits of AL-PS as a novel preventative target molecule for treatment of IBD.

## 1. Introduction

Inflammatory bowel disease (IBD) is characterized by chronic intestinal inflammation that occurs due to the interactions between genetic factors, host immune system and environmental factors [1]. Inflammation initiated by an immune response targets the T cells, macrophages, neutrophils, and other leukocytes producing cytokines, growth factors, neuropeptides, reactive oxygen metabolite, nitric oxide, and proteolytic enzymes, which accumulates at the site of inflammation [2,3]. Imbalance of inflammatory cytokines plays a major role in T cell dysregulation, resulting in the growth of uncontrolled hyperactive and auto reactive T cells. This creates an imbalance of Treg/Th1, Th2, and Th17 population in the activated state, which eventually contributes to the pathogenesis of IBD [4,5]. In IBD, pro-inflammatory cytokines, such as IL-1, tumour necrosis factor (TNF)-α, IL-6, and IL-17, are consistently enhanced [5,6]. Additionally, in some cases immunoregulatory cytokines, such as IL-10, can act as therapeutical agents in IBD [2,5].

At present, 5-aminosalicylic acid and its analogs, corticosteroids or non-specific immunosuppressive drugs are generally used in conventional therapies for IBD [7]. However, these conventional medical therapies exhibit limited efficacy and are associated with more serious complications and side-effects [8]. Anti-TNF-α antibody agents have been used as effective immune-modulatory drugs to treat severe cases of IBD [9]. However, some reports have shown that anti-TNF-α can induce skin lesions due to immunological imbalance [10,11]. Hence, dietary products that contain novel bioactive molecules, and modulate immune and inflammatory responses, may provide effective and safe clinical treatment options for IBD. Due to its safety and widespread occurrence in nature, an increasing body of evidence suggests that proteins and/or peptides from natural sources might be better suited for treatment of intestinal diseases. For instance, di- and tri peptides, derived from soy, were found to be readily absorbed *in vivo* [12] and demonstrated reduced myeloperoxidase (MPO) activity and down-regulation of TNF-α, IL-6, IFN-γ, IL-1β, and IL-17A gene expression in dextran sodium sulphate (DSS)-induced colitic pigs [13]. Others have identified protein or peptide based therapeutic effects via reduction of pro-inflammatory cytokines and increase in expression of anti-inflammatory mediators in DSS- or TNBS-induced colitis in animal models [14,15,16].

Eggshell membrane (ESM) is a natural material that is readily available and consists of about 64 proteins, which include Type I, V and X collagen, lysozyme, osteopontin, and sialoprotein [17,18]. It was shown earlier that ESM supplementation is a clinically valid therapy for joint and connective tissue disorders associated with inflammation [19,20]. *In vitro* and *in vivo* studies, have also demonstrated earlier that ESM-derived products can inhibit TNF-α production in peripheral blood mononuclear cells and suppress secretion of pro-inflammatory cytokines in serum of LPS-challenged rats [21,22]. Recently, we demonstrated that ESM hydro lysate had anti-oxidative stress activity and significantly reduced secretion of pro-inflammatory cytokine IL-8 in intestinal epithelial cells [23], which augmented that ESM hydro lysate, could serve as a potential anti-inflammatory supplement for intestinal health. Hence, an *in vivo* study was conducted to investigate the effects of ESM hydro lysate using DSS-induced colitic mice as a model and the results are presented in this paper.

## 2. Results and Discussion

### 2.1. Anti-Inflammatory Activity of AL-PS in Vitro

**Figure 1:**
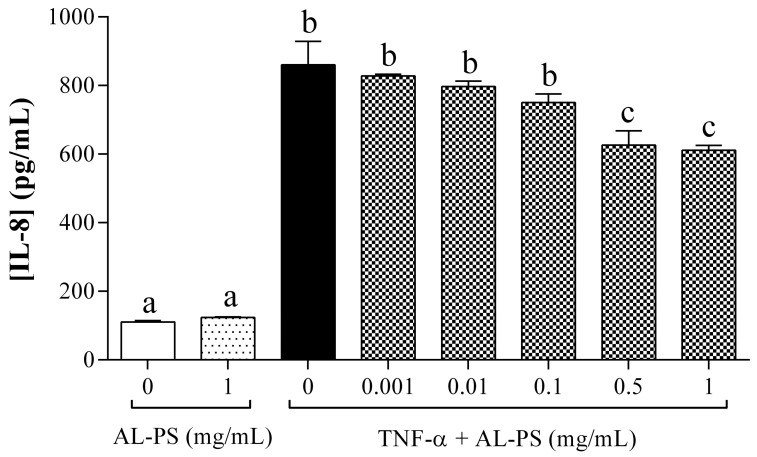
Effect of AL-PS on IL-8 secretion in TNF-α-induced Caco-2 cells. Cells were treated with different concentrations of AL-PS for 2 h and then stimulated with 2 ng/mL TNF-α for 4 h. Data are presented as mean ± SEM, *n* = 4. Experiments were repeated 3 times. Bars with different alphabet have mean values that are significantly different compared to cells treated with TNF-α alone (*p* < 0.05).

Previous studies showed that pro-inflammatory cytokine IL-8 can be produced in the cells after stimulation of TNF-α to induce acute inflammation [24]. To study the effects of AL-PS on IL-8 secretion, Caco-2 cells were treated with different concentration of AL-PS (0.001, 0.01, 0.1, 0.5, and 1 mg/mL) for 2 h and then stimulated with 2 ng/mL TNF-α for 4 h. As shown in Figure 1, AL-PS inhibited IL-8 secretion at 0.5 and 1 mg/mL, respectively (*p* < 0.05). AL-PS alone had no effect on IL-8 secretion.

### 2.2. Attenuation of Inflammation Signs in Vivo

**Figure 2:**
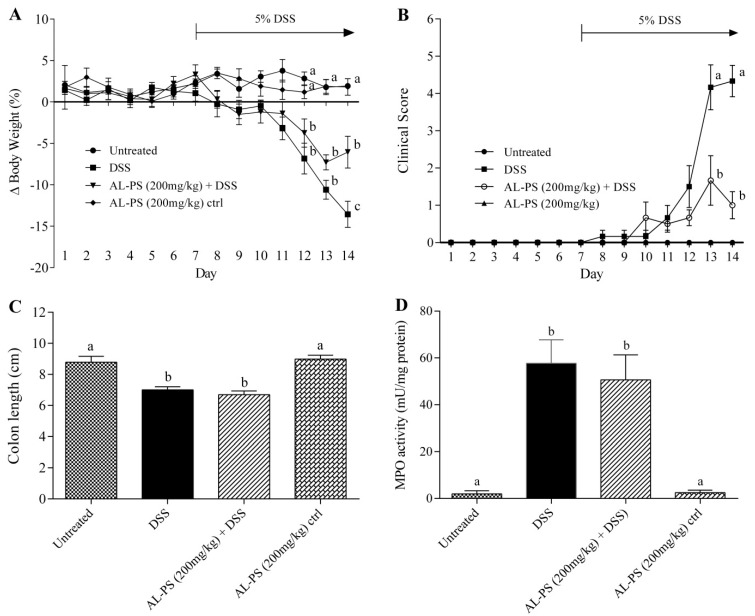
Effect of AL-PS on (**A**) daily weight loss; (**B**) daily clinical signs; (**C**) colon length; and (**D**) MPO activity in DSS-induced colitis. Mice were given 200 mg/kg BW of AL-PS daily for 14 days. From day 7, colitis was induced by 5% DSS in drinking water. Weight loss was calculated as percentage change relative to day 0. Colons were measured from the end of the cecum to the anus. Data represent means ± SEM of *n* = 6 mice/group. Different letters indicate statistically significant differences at *p* < 0.05. Unless indicated, no significant difference was observed between groups.

Anti-inflammatory effects of AL-PS were assessed using DSS-treated colitic mouse model *in vivo*. Significant weight loss was evident in the DSS-treated group compared to the negative control starting from day 12. In contrast, mice manifested less weight loss in the AL-PS treated group on day 14 (Figure 2A). Clinical signs of colitis, such as diarrhea, rectal bleeding, and lethargy, were observed in the positive control group after five days of DSS induction. Severity of clinical signs were significantly decreased (*p* < 0.05) in AL-PS-treated colitic mice when compared with the positive control group (Figure 2B). However, the colon length and MPO activity did not differ significantly (Figure 2C,D). Additionally, there was no effect of the administration of AL-PS alone on weight loss, colitis symptoms, colon length, or MPO activity (*p* > 0.05).

### 2.3. Cytokine Concentration

**Figure 3:**
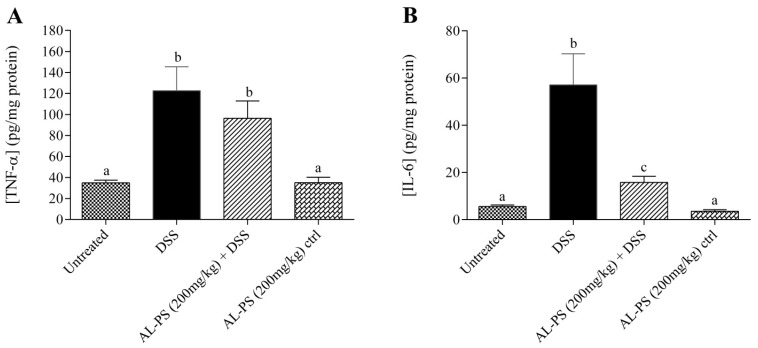
Effect of AL-PS on the concentration of (**A**) TNF-α and (**B**) IL-6 in DSS-induced colitis. Data represent means ± SEM of *n* = 6 mice/group. Different letters indicate statistically significant differences at *p* < 0.05.

Concentration of inflammatory cytokines TNF-α and IL-6 were measured. Treatment with 200 mg/kg of AL-PS inhibited the concentration of TNF-α and IL-6 in DSS-induced mice (Figure 3). IL-6 was reduced by 3.5-fold (*p* < 0.05) in AL-PS-treated colitic mice as compared to the positive control group. Administration of AL-PS alone at 14 days had no effect on secretion of TNF-α and IL-6 production in mice.

### 2.4. Gene Expression

**Figure 4:**
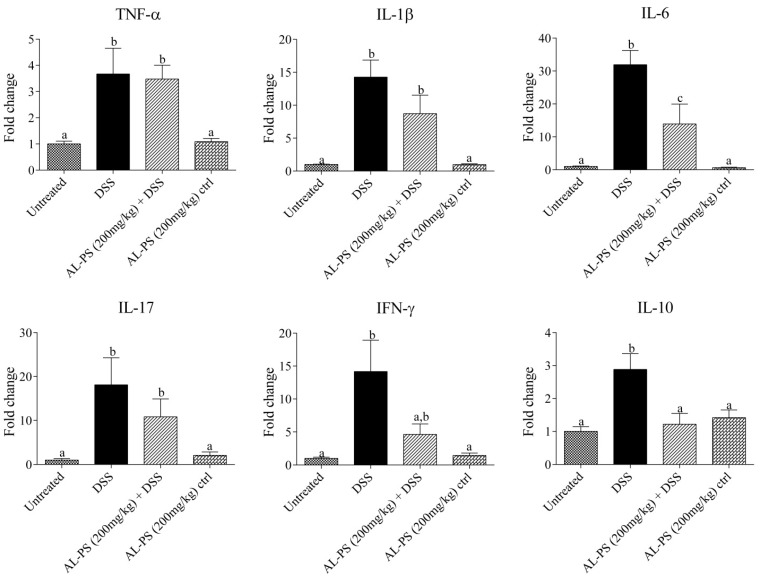
Effect of AL-PS on gene expression of pro-inflammatory and anti-inflammatory cytokines in DSS-induced colitis. RNA was isolated from colonic tissue and relative mRNA expression of TNF-α, IL-1β, IL-6, IL-10, IL-17, and IFN-γ were measured by real-time RT-PCR. Results are reported as fold change relative to negative control (untreated) animals and data represent means ± SEM of *n* = 6 mice/group. Different letters indicate statistically significant differences at *p* < 0.05.

**Figure 5:**
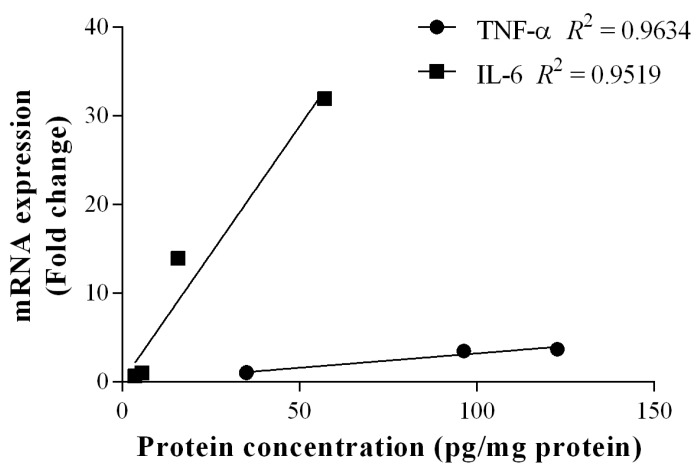
Correlations between proteins and gene expressions of TNF-α and IL-6. Protein concentration of TNF-α was correlated with its mRNA expression, Pearson’s correlation coefficient: r = 0.98, *R*^2^ = 0.96, *p* < 0.05. Protein concentration of IL-6 was correlated with its mRNA expression, Pearson’s correlation coefficient: r = 0.98, *R*^2^ = 0.95, *p* < 0.05.

**Figure 6:**
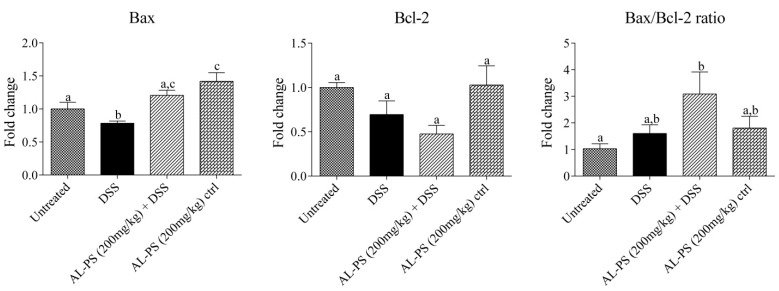
Effect of AL-PS on gene expression of (apoptosis promoter) and *Bcl-2* (apoptosis inhibitor), and *Bax/Bcl-2* ratio in DSS-induced colitis. RNA was isolated from colonic tissue and relative mRNA expression was measured by real-time RT-PCR. Results are reported as fold change relative to negative control (untreated) animals and data represent means ± SEM of *n* = 6 mice/group. Different letters indicate statistically significant differences at *p* < 0.05.

Anti-inflammatory effects of AL-PS with regards to local mRNA expression of inflammatory cytokines were performed. Administration of 200 mg/kg AL-PS for all tested inflammatory cytokine mRNA expression resulted in a trend towards decrease in expression when compared with positive control mice (Figure 4). Reduction of IL-6 expression in AL-PS-treated colitic mice was statistically significant (*p* < 0.05). The relationship between protein level and gene expression of TNF-α or IL-6 was assessed by Pearson’s correlation analysis. As shown in Figure 5, there was a significant positive correlation between protein level and gene expression of TNF-α (r = 0.98, *p* < 0.05) and IL-6 (r = 0.98, *p* < 0.05). In addition, the expression of IL-10 was increased in the positive control group and was decreased significantly (*p* < 0.05) in AL-PS-treated colitic mice as compared to the positive control mice. The treatment with AL-PS alone had no effect on mRNA gene expression. Expression of pro-apoptotic *Bax* gene was significantly increased (*p* < 0.05) with 200 mg/kg of AL-PS administration in colitic mice when compared with the positive control group (Figure 6). However there was no change with the anti-apoptotic *Bcl-2* gene and with *Bax/Bcl-2* ratio between groups (*p* ˃ 0.05). There is a trend towards an increase with the *Bax/Bcl-2* ratio in the AL-PS treated group as compared to the positive control group.

### 2.5. Discussion

TNF-α a pro-inflammatory cytokine produced by activated macrophages and monocytes plays a crucial role in up-regulating Th1-dependent inflammatory response in the gut, resulting in mucosal inflammation in case of IBD [21]. Here, we observed that AL-PS reduced TNF-α-induced IL-8 secretion *in vitro*. IL-8 was decreased significantly with the treatment of AL-PS at the concentration of 0.5 or 1 mg/mL, which indicates that AL-PS exhibited beneficial effect in the early stage of inflammation. *In vitro*, reduction of TNF-α-associated expression of the IL-8 cytokine by Caco-2 cells has been linked to inhibition of NF-κB activation, and was found to be correlated with *in vivo* anti-inflammatory activity in mice [22]. Hence, effects of anti-inflammatory activity of AL-PS was studied in DSS-induced colitic mouse model. It was earlier shown that administration of DSS in mice led to body weight loss, epithelial cell inflammation, mucosal ulceration, neutrophil infiltration, colon shortening and bloody diarrhea [2,25,26] which were similar to the signs as observed with ulcerative colitis (UC). However, in this study we found that AL-PS showed significant protective effects to ameliorate weight loss and clinical signs of colitis in DSS-induced mice as compared to mice given DSS alone. These results were similar to those reported previously in which peptides from soy proteins reduced the severity of inflammation in DSS-induced mice [13,27]. These results indicate that the oral administration of AL-PS ameliorated severity of DSS-induced colitis in mice. Comparison of histopathological scoring and H&E imaging of colonic tissues could be scope for future research to further characterize the ability of the eggshell membrane hydro lysate in ameliorating inflammation.

Both TNF-α and IL-6, as critical pro-inflammatory cytokines, are recognized as mediators of inflammation in IBD. TNF-α is involved in the enhancement of IL-1β and IL-6, expression of adhesion molecules, and inhibition of apoptosis [5]. In this study, treatment with AL-PS inhibited the concentration of IL-6 as compared to positive control group. IL-6 induces STAT-3 activation which mediates resistance against apoptosis; IL-6 also induces NF-κB activation to enhance the expression of adhesion molecule in IBD [6]. Recent evidence on anti-IL-6 or anti-IL-6 receptor targets has focused on developing promising therapeutic approaches to abrogate intestinal inflammation and clinical symptoms of IBD. Blockade of IL-6 signaling has been reported to be effective in prevention of chronic intestinal inflammation in animals and human [6,28]. These results validate the therapeutic potential of AL-PS supplementation in intestinal inflammation.

Intestinal inflammation is mediated by T cells, such as Th1, Th2, or Th17 which occurs due to dysregulation or imbalance in T-cell immune homeostasis and induction of chronic intestinal inflammation [5,29]. Production of many pro-inflammatory cytokines is known to increase with IBD, and high level of pro-inflammatory cytokines in the damaged tissue results in increased inflammation. All inflammatory cytokine mRNA gene expression AL-PS treated groupresulted in a trend towards decrease in expression when compared with positive control mice. Increased secretion of TNF-α, IL-1β, IL-6, and IL-17 is known to play prominent role in the early stages of inflammation and is known to promote inflammatory reaction [5,6,29,30,31]. TNF-α and IL-1β have been reported to be up-regulated in DSS-induced colitic mice [32]. IFN-γ has been found to change the protein and lipid composition of the membrane micro domains of epithelial cell tight junctions, which leads to chronic intestinal inflammation and mucosal injury [33,34]. It is also noteworthy that in this study, significant reduction of IL-6 gene expression in AL-PS-treated colitic mice positively correlated to those of IL-6 protein concentration as determined by ELISA. IL-6 is produced by both T cells and macrophages and is involved in Th1 and Th17 cell response. IL-6 exerts its pro-inflammatory effects via IL-6 trans-signaling, through the soluble form of the IL-6 receptor and this results is the induction of anti-apoptotic genes and subsequent resistance of T cells to apoptosis, leading to uncontrolled expansion of activated T cells [6]. According to Pearson’s correlation analysis performed, gene expression of TNF-α and IL-6 was shown to be positively correlated to protein concentration measured. These results indicated that the effect of AL-PS may be mediated by IL-6-cell signaling pathway thereby inhibiting inflammation in colitic mice. In addition, the concentration of anti-inflammatory cytokine IL-10 was significantly decreased in the AL-PS-treated mice as compared to positive control. Similar trend has been earlier reported in experimental animal models and IBD patients suffering from chronic inflammation [27,35,36]. Many studies have shown that anti-inflammatory cytokines can modulate the amplification of inflammation [5]. IL-10 plays a crucial role in inhibition of antigen presentation and subsequent release of pro-inflammatory cytokines, thereby maintaining mucosal homeostasis and regulating colonic inflammation during experimental colitis [36,37]. Overall, these results indicate that AL-PS may have anti-inflammatory activity in DSS-induced colitis by inhibiting the expression of pro-inflammatory cytokines rather than enhancing the expression of anti-inflammatory mediators.

Since IL-6 trans-signaling is related to apoptosis and IL-6 exerts cytoprotective effects by promoting the expression of proteins Bcl-2 and Bax. The expression of Bax and Bcl-2 was determined with regards to AL-PS restoring susceptibility of activated T cells in apoptosis. The trend of increasing expression of pro-apoptotic *Bax* gene suggests that AL-PS may play a role in restoring intestinal immune homeostasis. Similar results were observed in tryptophan-treated colitic porcine model [38]. *Bax/Bcl-2* ratio resulted in a trend towards increase in case of AL-PS-treated colitic mice. As known earlier, the DSS-induced inflammation in colon is similar to what is observed with UC [25]. It was earlier reported that the amount of Bcl-2^+^ cells in UC mucosa was fewer than those of the normal mucosa and most importantly the *Bax/Bcl-2* ratio was higher in UC compared with control musosa [39], suggesting apoptosis of T cells in DSS-induced intestinal inflammation. These results validate that AL-PS administration may ameliorate DSS-induced clinical signs and weight loss by facilitating apoptosis of activated T cells and aiding in restoring gut homeostasis.

In conclusion, AL-PS effectively inhibited IL-8 secretion *in vitro* and showed protective effects against DSS-induced inflammation *in vivo*. Weight loss and clinical signs were ameliorated after treatment with AL-PS. This study thereby concludes that AL-PS ameliorates inflammation in DSS induced colitic mice model via IL-6-mediated pathway and promotes T cells apoptosis to restore immune homeostasis in gut. Further research could focus on the specific mechanism of IL-6 pathway regulated by AL-PS.

## 3. Experimental Section

### 3.1. Materials

All reagents were purchased from Sigma-Aldrich Co. (St. Louis, MO, USA) unless or otherwise specified.

### 3.2. Preparation of ESM Hydro Lysate

ESM hydrolysate (AL-PS) was prepared by digestion of ESM using a combination of Alcalase (EC 3.4.21.62; HBI Enzymes Inc., Osaka, Japan) and Protease S (Amano Enzymes, Nagoya, Japan) enzymes according to a method previously described [23]. Briefly, ESM was digested with Alcalase (1%, *w*/*w*) at 55 °C for 4 h, and then Protease S (2%, *w*/*w*) was added into the mixture for another 12 h. Enzymes were deactivated at 80 °C for 20 min. After centrifugation, the supernatant was collected, lyophilized, and stored at −20 °C until further use.

### 3.3. Cell Culture

The Caco-2 human intestinal cell line (American Type Culture Collection, Rockville, MD, USA) was used in this study. The cells were cultured in growth medium (Dulbecco’s modified Eagle’s medium/F12) (DMEM/F12; Gibco, Burlington, ON, Canada) with 20% foetal bovine serum (FBS; Hyclone, UT, USA) and 50 units/mL of penicillin-streptomycin (Gibco), and incubated at 37 °C in 5% CO_2_. Cell passage numbers 20–40 were used. The cells were grown in a flask for 5–7 days with fresh medium replaced every 2 days.

### 3.4. In Vitro Induction of Inflammation

Caco-2 cells were seeded at concentration of 2 × 10^5^ cells/mL in a 48-well plate (Corning Costar, Cambridge, MA, USA) and grown for 7–10 days with fresh medium replaced every 2 days. AL-PS was added at different concentrations into cells grown in DMEM/F12 with 5% FBS and incubated for 2 h at 37 °C in 5% CO_2_. Then, 2 ng/mL TNF-α (Gibco) was added to the cells and incubated for an additional 4 h. Cell supernatants were then collected and stored at −80 °C until use.

### 3.5. Determination of IL-8 Secretion

The concentration of IL-8 was quantified by ELISA as according to a previously described method [23]. The concentration of IL-8 was detected using purified mouse anti-human IL-8 monoclonal antibody (1:1000 dilution, *v*/*v*, BD Biosciences, San Diego, CA, USA) and biotinylated mouse anti-human IL-8 antibody (1:2000 dilution, *v*/*v*, BD Biosciences). The standard curves for IL-8 was established using serial dilutions of recombinant IL-8 standards (BD Biosciences). The optical density was measured at 450 nm using a micro plate reader (Bio-Rad model 550, Bio-Rad Laboratories, Hercules, CA, USA).

### 3.6. Animals and Experimental Design

Female Balb/c mice (18–20 g; Charles River Laboratories, Inc., Montreal, QC, Canada) were housed on a 12-h light-dark cycle and had unlimited access to standard mouse chow and water. Following a seven-day acclimatization period, mice were given AL-PS (200 mg/kg body weight; BW) every day for 14 days by oral gavage. On day 7, 5% (*w*/*v*) DSS (36–50 kDa; MP Biomedicals, Solon, OH, USA) was added to the drinking water to induce acute colitis. Mice were weighed daily and evaluated for clinical signs of colitis. Control mice received water only. To evaluate the effect of the peptide alone, one group received peptide (high dose, 200 mg/kg) alone without DSS.

After 14 days, mice were euthanized, and colons were excised and measured, and sectioned for further analysis. All animal experiments were conducted in accordance with the Canadian Council of Animal Care Guide for the Care and Use of Experimental Animals and were approved by the Animal care Committe, University of Guelph (AUP 07R113; eAUP No. 1536; Approval date: 2 August 2011).

### 3.7. Clinical Evaluation of Colitis

Mice were weighed daily and inspected for stool consistency, presence of blood in stool or bleeding, and general appearance. Clinical scoring was carried out as described previously [40], and is defined as follows. Stool score: 0 = normal; 1 = moist/sticky stool; 2 = soft stool; 3 = diarrhea. Stool blood score: 0 = no blood; 1 = evidence of blood in stool or around anus; 2 = severe bleeding. Mouse appearance: 0 = normal; 1 = ruffled fur or altered gait; 2 = lethargic or moribund.

### 3.8. Determination of TNF-α and IL-6 Concentration in Tissue

Colon tissues were rinsed with PBS containing 1mM phenylmethylsulfonyl fluoride (PMSF), and flash frozen using liquid nitrogen and stored at −80 °C until further use. Tissues were homogenized in a mixture of 10 µg/mL aprotinin, 10 µg/mL leupeptin, and 10 µg/mL pepstatin A in PBS containing 1 mM PMSF. Homogenates were separated by centrifugation at 12,000 rpm for 15 min at 4 °C. TNF-α and IL-6 concentrations were measured by ELISA, according to the manufacturer’s guidelines (BD Biosciences, Minneapolis, MN, USA). Protein concentration was measured by DC Protein assay (Bio-Rad Laboratories, Inc., Hercules, CA, USA). TNF-α and IL-6 concentrations are expressed as pg cytokine per mg protein.

### 3.9. Determination of MPO Activity

Tissues were homogenized in 50 mM potassium phosphate, pH 6.0 containing 0.5% hexadecyl-trimethyl-ammonium bromide (HTAB), and subjected to freeze-thaw procedure twice. Samples were separated by centrifugation at 12,000 rpm for 10 min at 4 °C, and supernatants were assayed for MPO activity and protein concentration was measured by DC Protein assay (Bio-Rad Laboratories, Inc.). Samples were diluted in HTAB buffer and mixed with a 1.6 mM 3,3',5,5'-tetramethylbenzidine (TMB) in 0.3 mM H_2_O_2_. One unit of enzyme activity was defined as the amount of MPO present that caused a change in absorbance of 1.0/min at 655 nm. MPO amounts are given as mU MPO per mg protein.

### 3.10. RNA Isolation and Determination of Gene Expression in the Colon

Tissues were rinsed in PBS containing 1 mM phenylmethylsulfonyl fluoride (PMSF) and stored in RNAlater (Ambion/Life Technologies, Burlington, ON, Canada). Total RNA was extracted using the Aurum™ Total RNA Mini Kit (Bio-Rad Laboratories, Inc., Hercules, CA, USA) according to the manufacturer’s instructions. First-strand cDNA synthesis was carried out using the qScript™ cDNA Synthesis Kit (Quanta Biosceinces, Inc., Gaithersburg, MD, USA) according to the manufacturer’s instructions. Real-time PCR was carried out using PerfeCTa^®^ SYBR^®^ Green Supermix (Quanta Biosceinces, Inc.) on a MyiQ™ Single Color Real-Time PCR Detection System (Bio-Rad Laboratories, Inc.) using the following conditions: denaturation 15 s at 95 °C, annealing 15 s at 56 °C, and extension 30 s at 72 °C. Primers listed in Table 1 were designed using Primer3 v.0.4.0 [41] and synthesized by the University of Guelph Laboratory Services Molecular Biology Section. Relative gene expression was calculated by 2^−ΔΔ*C*t^ method [42] using glyceraldehyde-3-phosphate dehydrogenase (*GAPDH*) as the reference gene. Results are expressed as fold change relative to the negative control (untreated) animals.

**Table 1:** Mouse primer pairs used for real-time PCR.

Accession No.	Gene	Forward Primer (5'-3')	Reverse Primer (5'-3')
NM_008084	*GAPDH*	AACTTTGGCATTGTGGAAGG	GGATGCAGGGATGATGTTCT
NM_013693	*TNF-α*	CCCCAAAGGGATGAGAAGTT	CACTTGGTGGTTTGCTACGA
NM_008361	*IL-1β*	GGATGAGGACATGAGCACCT	AGCTCATATGGGTCCGACAG
NM_031168	*IL-6*	CCGGAGAGGAGACTTCACAG	CAGAATTGCCATTGCACAAC
NM_010548	*IL-10*	GCCTTATCGGAAATGATCCA	AGGGGAGAAATCGATGACAG
NM_010552	*IL-17A*	CCAGGGAGAGCTTCATCTGT	AGGAAGTCCTTGGCCTCAGT
NM_008337	*IFN-γ*	GCTCTTCCTCATGGCTGTTT	GTCACCATCCTTTTGCCAGT
NM_007527	*Bax*	GTGAGCGGCTGCTTGTCT	GGTCCCGAAGTAGGAGAGGA
NM_009741	*Bcl-2*	TGGATCCAGGATAACGGAAG	CAAACAGAGGTCGCATGCTG

### 3.11. Statistical Analysis

Results were expressed as mean ± SEM. Statistical analyses were carried out using GraphPad software (GraphPad, San Diego, CA, USA). The statistical significance of the data was determined by one-way ANOVA followed by Tukey’s multiple-comparison test. Pearson’s correlation coefficient was used to determine the relationship between the indicators. *p* ≤ 0.05 was taken as value of significance.

## 4. Conclusions

In conclusion, this study validates possible mechanism of anti-inflammatory activity of AL-PS derived from ESM *in vivo*. Administration of AL-PS ameliorated DSS-induced colitis signs and severity of inflammation in mice via down-regulation of pro-inflammatory cytokine expression and boosting T cells apoptosis to restore immune homeostasis in gut, suggesting that AL-PS may be a promising preventative target for curing intestinal inflammation, such as IBD. Though there is limitation in current study, it has important implication for providing with a solution to use eggshell membrane as high value-added material to produce preventative products with anti-inflammatory activity.
